# Nutrient allocation strategies of woody plants: an approach from the scaling of nitrogen and phosphorus between twig stems and leaves

**DOI:** 10.1038/srep20099

**Published:** 2016-02-05

**Authors:** Zhengbing Yan, Peng Li, Yahan Chen, Wenxuan Han, Jingyun Fang

**Affiliations:** 1 Department of Ecology, College of Urban and Environmental Sciences, and Key Laboratory for Earth Surface Processes of the Ministry of Education, Peking University, Beijing, China 100871; 2 Institute of Botany, Chinese Academy of Sciences, Beijing, China 100093; 3 College of Resources and Environmental Sciences, Beijing Key Laboratory of Biodiversity and Organic Farming, China Agricultural University, Beijing, China 100193

## Abstract

Allocation of limited nutrients, such as nitrogen (N) and phosphorus (P), among plant organs reflects the influences of evolutionary and ecological processes on functional traits of plants, and thus is related to functional groups and environmental conditions. In this study, we tested this hypothesis by exploring the stoichiometric scaling of N and P concentrations between twig stems and leaves of 335 woody species from 12 forest sites across eastern China. Scaling exponents of twig stem N (or P) to leaf N (or P) varied among functional groups. With increasing latitude, these scaling exponents significantly decreased from >1 at low latitude to <1 at high latitude across the study area. These results suggested that, as plant nutrient concentration increased, plants at low latitudes showed a faster increase in twig stem nutrient concentration, whereas plants at high latitudes presented a faster increase in leaf nutrient concentration. Such shifts in nutrient allocation strategy from low to high latitudes may be controlled by temperature. Overall, our findings provide a new approach to explore plant nutrient allocation strategies by analysing the stoichiometric scaling of nutrients among organs, which could broaden our understanding of the interactions between plants and their environments.

Allocation of limited nutrients, as an important strategy for plants in response to the changing environments, reflects the influences of evolutionary and ecological processes and trade-offs of multiple functions^1^,^2^,^3^,^4^. To maximize plant growth and maintain the optimal metabolic activities, plants need to balance the allocation of nutrients across organs under different stresses^1^,^4^,^5^,^6^,^7^. For example, plants under low soil nutrient availability often translocate nutrients from senesced tissues to new leaves because of the important carbon gain^5^. Plants under arid conditions generally allocate more N to leaves to compensate the low photosynthetic rate induced by the reducing stomatal conductance^8^,^9^. However, current knowledge of plant nutrient allocation strategies mainly comes from herbs, shrubs and tree seedlings^1^,^7^, with little knowledge from forest trees.

Exploring the associations of nutrient content across organs may be a way to reveal the allocation strategies of nutrients for tree species. From the ontogenetic perspective, plant nutrient contents across organs are tightly coordinated, and their associations can be examined by scaling relationship analyses^2^,^6^,^7^,^10^. Scaling relationships among plant traits are widely studied, mainly in two ways: relationship of different traits within a specific organ and relationship of the same traits among organs. Within a specific organ, many scaling relationships are observed, such as leaf N *vs.* leaf P^2,^^11^,^12^,^13^, leaf lifespan *vs.* leaf mass per area^14^, nutrient content *vs.* photosynthetic rate^12^, and nutrient content *vs.* respiration rate^15^. For instance, the scaling exponent of leaf N to leaf P is <1, indicating a decline in leaf N:P with increasing plant growth rate, consistent with the “growth rate hypothesis”^2^,^11^,^12^,^16^. Among organs, previous studies focused on biomass partitioning^17^, and morphological linkages^18^,^19^,^20^. For example, Enquist and Niklas (2002)^17^ reported that leaf biomass scaled as the 3/4 power of stem biomass in seed plants, showing that plants allocate more biomass to stems than to leaves across a large scale. By contrast, the understanding of scaling relationships of nutrients among organs, especially for woody plants, is very limited.

Leaves of vascular plants play a crucial role in conducting photosynthesis, whereas twig stems provide mechanical support and transport water, carbohydrates and nutrients^21^,^22^,^23^. N and P allocated to leaves are the key components of metabolic and photosynthetic apparatuses^24^, whereas N and P allocated to twig stems play important roles in respiration^15^, internal nutrient recycling^1^,^25^, and photosynthate loading and export in the phloem^24^. Owing to the functional linkages between these two organs, nutrient concentrations between leaves and twig stems should also have tight associations via plant nutrient allocation strategies. Two recent studies revealed that scaling exponents of stem N (or P) to leaf N (or P) were >1, likely because plants required higher nutrient investments in stems (for photosynthate loading and export in the phloem^24^) than in leaves as plant nutrient increased^2^,^7^.

Like plant functional traits (e.g., leaf N, leaf P, leaf mass per area or leaf lifespan), the allocation of nutrients among organs can be considered as another plant trait that may reflect the interactions between plants and their environments *per se*. Previous studies indicate that functional groups, climate and soils have significant influences on plant functional traits, and jointly determine their biogeographic patterns^14^,^23^,^26^,^27^,^28^,^29^,^30^. Therefore, we hypothesize that nutrient allocation among plant organs varies among functional groups and changes with environmental factors, causing a unique biogeographic pattern. Specifically, we hypothesize that: (1) as plant nutrient concentration increases, there is a faster increase in twig stem nutrient concentration for evergreen plants relative to deciduous plants and for legume relative to non-legume; (2) as plant nutrient concentration increases, plants at low latitudes show a faster increase in twig stem nutrient concentration, whereas plants at high latitudes show a faster increase in leaf nutrient concentration; and (3) both functional group and temperature together drive the latitudinal pattern of nutrient allocation, whereas soil nutrient availabilities and precipitation play modest roles in determining this pattern. In this study, we are to test these hypotheses by examining how functional groups, climate and soils jointly control the stoichiometric scaling of twig stem N (or P) to leaf N (or P) of 335 woody species from 12 forest sites in eastern China.

## Results

### Patterns of scaling exponents across functional groups

**Figure 1:**
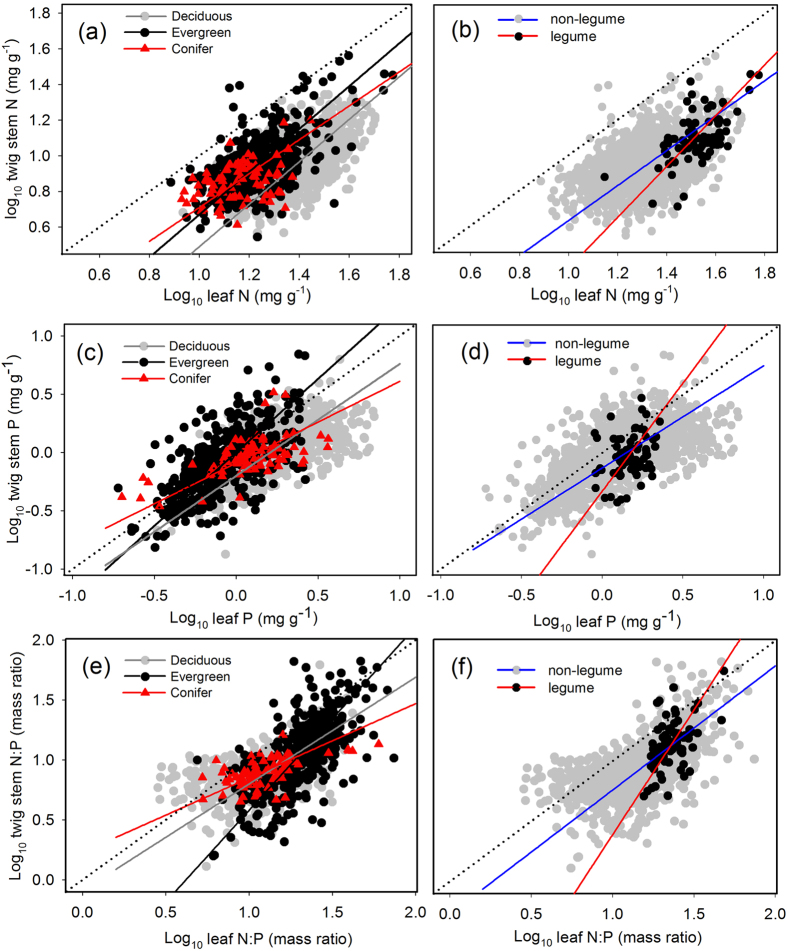
Scaling relationships of twig stem N (or P, or N:P ratio) to leaf N (or P, or N:P ratio) for woody plants by functional group (deciduous/evergreen/conifer; legume/non-legume). Reduced major axis (RMA) regression was used to determine the significant line (*p* < 0.05). All data were log_10_-transformed before analysis.

There were significant stoichiometric scaling relationships between twig stem N (or P, or N:P ratio) and leaf N (or P, or N:P ratio), but their scaling exponents varied among functional groups (Table 1; Fig. 1). Scaling exponents of twig stem P to leaf P (*α*_P_) and twig stem N:P ratio to leaf N:P ratio (*α*_N:P_) were highest in evergreen broad-leaved plants, followed by deciduous broad-leaved and coniferous plants (1.26, 0.96 and 0.70 for *α*_P_; 1.53, 0.89 and 0.62 for *α*_N:P_), whereas scaling exponent of twig stem N to leaf N (*α*_N_) was similar among the three functional types (1.20, 1.19 and 0.95). Legume species had higher scaling exponents than non-legume species (1.44 *vs.* 0.99 for *α*_N_; 1.86 *vs.* 0.88 for *α*_P_; 2.07 *vs.* 1.03 for *α*_N:P_).

### Relationships between scaling exponents and latitude/environmental factors

**Figure 2:**
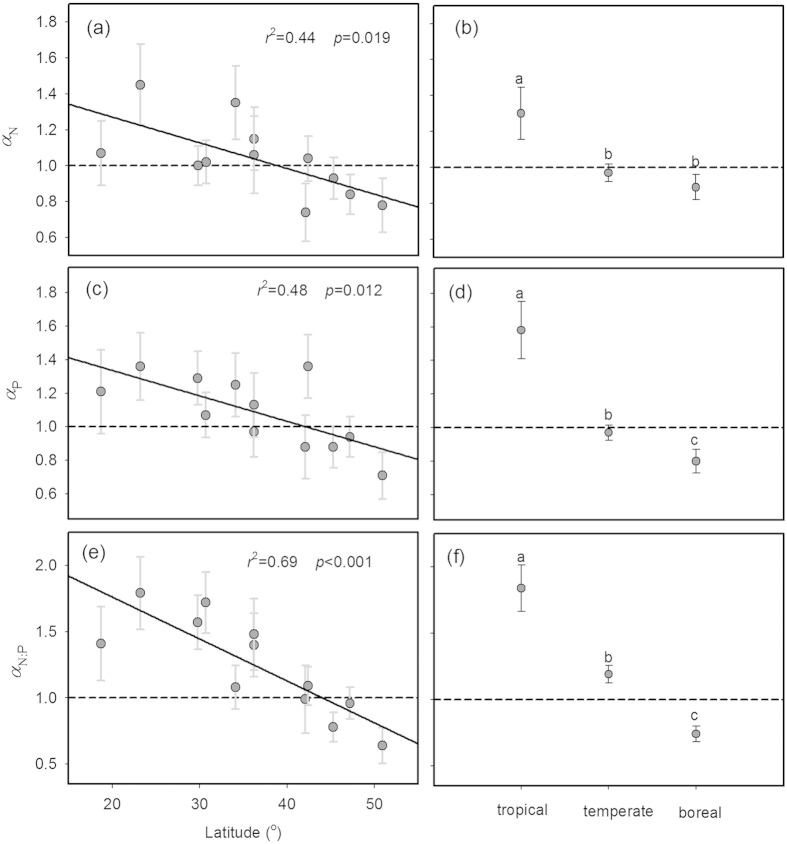
The scaling exponents, *α*_N_, *α*_P_ and *α*_N:P_, in tropical, temperate and boreal forests and along the latitudinal gradients in eastern China. Points and error bars show the exponents and 95% confidence interval (CI). Different letters indicate significant difference (*p* < 0.05) based on a likelihood ratio test. Significant (*p* < 0.05) regression lines are fit to the exponents.

**Figure 3:**
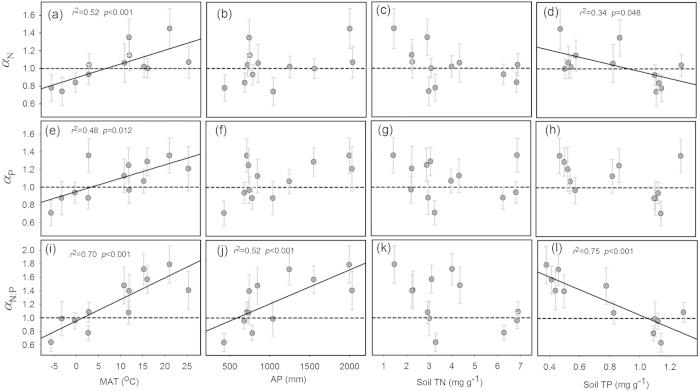
Relationship between scaling exponents (*α*_N_, *α*_P_ and *α*_N:P_) and environmental factors. Points and error bars show the exponent and 95% confidence interval (CI), and significant regression lines (*p* < 0.05) are fit to the exponents.

As latitude increased, scaling exponents for N and P and N:P ratio significantly decreased from >1 at low latitude (23.2°N) to <1 at high latitude (50.9°N), and this trend was consistent among forest types (i.e., tropical, temperate and boreal forest) (Fig. 2; Table S2 and S3). Along the latitudinal gradient, *α*_N_ ranged from 1.45 at Mt. Dinghu with 23.2°N to 0.74 at Mt. Changbai with 42.1°N, and *α*_P_ varied from 1.36 at Mt. Dinghu to 0.71 at Mt. Genhe with 50.9°N, and *α*_N:P_ varied from 1.79 at Mt. Dinghu to 0.64 at Mt. Genhe with 50.9°N. Across the three biomes, *α*_N,_ *α*_P_ and *α*_N:P_ were highest in tropical forests, followed by temperate and boreal forests (1.30, 0.97 and 0.89 for *α*_N_; 1.58, 0.97 and 0.80 for *α*_P_; 1.84, 1.19 and 0.74 for *α*_N:P_) (Fig. 2; Table S2). Moreover, both *α*_N_ and *α*_P_ were significantly correlated with mean annual temperature (MAT) (*p* < 0.05), but weakly correlated with annual precipitation (AP), soil total N (TN) and total P (TP) concentrations (Fig. 3). Specifically, scaling exponents increased with increasing MAT, ranging from 0.74 at −3.3 °C to 1.45 at 21 °C for *α*_N_, and from 0.71 at −5.7 °C to 1.36 at 21 °C for *α*_P_. By contrast, *α*_N:P_ was significantly correlated with MAT and AP and soil TP, but weakly correlated with soil TN (Fig. 3i–l).

## Discussion

The higher *α*_P_ in evergreen broad-leaved species than in deciduous broad-leaved species (1.26 vs. 0.96 in Table 1) indicates that, as plant P increases, evergreen broad-leaved plants have a faster increase in twig stem P, whereas deciduous broad-leaved plants exhibit a faster increase in leaf P. Evergreen plants, distributed at low and mid-latitudes with low soil nutrient availability (Fig. S1)^31^, have evolved multiple adaptions to their environments, such as longer leaf lifespan, higher leaf mass per area and lower leaf photosynthetic rate than deciduous plants^14^,^32^,^33^,^34^. Compared with deciduous broad-leaved plants, evergreen broad-leaved plants might require more investments in phloem loading and export apparatus to meet the higher demand for photosynthate transport^1^,^31^, which is associated with increasing allocation of nutrients to twig stems. Additionally, their demand for nutrient storage via twig stems for a longer leaf lifespan might also induce a higher investment in twig stems than in leaves. In contrast, deciduous broad-leaved plants with short leaf lifespan need to maximize leaf photosynthetic activity to exploit light availabilities during the shorter growing season, resulting in more nutrient allocation to leaves. Furthermore, different from *α*_P_, *α*_N_ is similar between evergreen broad-leaved plants and deciduous broad-leaved plants (1.20 vs 1.19 in Table 1, *p* > 0.05), which indicates that the two functional types perform the common allocation strategy of N between twig stem and leaf. We infer that, compared with N, plants tend to change their P allocation strategies more easily across the functional group.

For both *α*_N_ and *α*_P_, legume species have higher values than non-legumes (1.44 *vs.* 0.99 for *α*_N_; 1.86 *vs.* 0.88 for *α*_P_) (Table 1), meaning that legume species allocate more N and P to twig stems as plant nutrient concentrations increase. Symbiotic N_2_-fixing bacteria in roots of legume species require much carbohydrate from photosynthetic tissues, because N acquisition through biological fixation has a higher energy cost than direct N absorption from the soil^3^,^35^. Thus, given the higher carbohydrate demand of symbiotic N_2_-fixing bacteria, legumes should increase the photosynthate loading and export rate in the phloem, which is associated with higher N and P investments in twig stems^2^.

**Figure 4:**
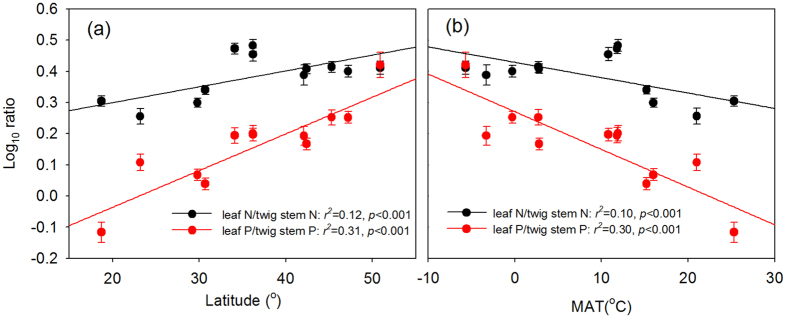
Relationship between ratios of leaf N (or P): twig stem N (or P) (leaf N/twig stem N; leaf P/twig stem P) and latitude (a) and MAT (b) Points and error bars show the means and standard errors, and significant (*p* < 0.05) regression lines are fit to the raw data.

**Figure 5:**
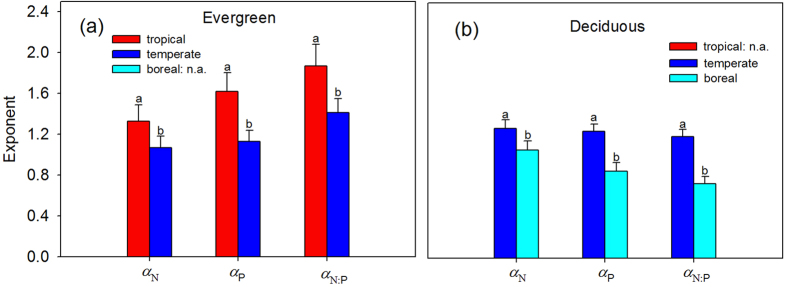
The scaling exponents, *α*_N_, *α*_P_ and *α*_N:P_, for evergreen broadleaved plants in tropical and temperate forests (a) and for deciduous broadleaved plants in temperate and boreal forests (b) Points and error bars show the exponent and 95% confidence interval (CI). Different letters indicate significant difference (*p* < 0.05) based on a likelihood ratio test.

Both *α*_N_ and *α*_P_ decrease from >1 to <1 with increasing latitude (Fig. 2), suggesting that, as plant nutrient concentration increased, plants at low latitudes tended toward a higher increase in twig stem nutrient concentration, whereas plants at high latitudes tended toward a higher increase in leaf nutrient concentration. This indicates a gradual shift in nutrient allocation strategy across a large geographic area. Moreover, plants at high latitudes had higher ratios of leaf N (or P) : twig stem N (or P) (Fig. 4a). These latitudinal patterns of scaling exponents (*α*_N_ and *α*_P_) and nutrient ratios (leaf N/P : twig stem N/P) may be attributed to the changes in species composition from evergreen broad-leaved plants at low latitudes to deciduous broad-leaved and coniferous plants at high latitudes (Fig. S2)^13^, because *α*_P_ was highest in evergreen broad-leaved plants, followed by deciduous broad-leaved and coniferous plants (1.26; 0.96; 0.70 in Table 1). However, the latitudinal pattern of *α*_N_ was hardly explained by the changes in species composition because of similar *α*_N_ across the three functional types. For evergreen broad-leaved plants, both *α*_N_ and *α*_P_ were higher in tropical forests than in temperate forests, and for deciduous broad-leaved plants, both *α*_N_ and *α*_P_ were higher in temperate forests than in boreal forests (Fig. 5). Thus, changes in species composition may play a modest role in latitudinal patterns of nutrient allocation. Note that in this study we did not conduct detailed analyses on the scaling exponents of evergreen needle plants at boreal forests and deciduous plants at tropical forests, and variations in the scaling exponents of coniferous plants among biomes because of the paucity of data and less representatives (Fig. 5; Fig. S2).

Through the exploration of the relationships between scaling exponents and environmental factors, we found that MAT could be a main driver for these latitudinal patterns because of the tightest correlation between MAT and latitude (*r*^2^ = 0.94, *p* < 0.001 in Table S4). As the temperature increased, scaling exponents increased from <1 to >1, meaning that, as plant nutrient concentration increased, plants at low temperature exhibited a higher increase in leaf nutrient concentration, whereas a higher increase in twig stem nutrient concentration occurred for plants at high temperature. Plants at low temperature also had higher ratios of leaf N (or P) : twig stem N (or P) (Fig. 4b). Previous studies have revealed that temperature is the main driver of biogeographic patterns of leaf N and P concentration^27^,^30^,^36^, and biomass allocation^37^. According to the “temperature-plant physiological hypothesis”^27^, low temperature induces an increase in leaf nutrient concentrations to offset diminished efficiency of N-rich enzymes and P-rich RNA. However, compared with leaves, twig stems are less influenced by temperature, because their optimal functions are less dependent on nutrient compositions^6^. Thus, more nutrients allocated to leaves at low temperature are probably an adaptive strategy for plants to maintain the functional equilibrium for fitness. Furthermore, temperature largely drives the changes in growing season length and leaf lifespan^34^, which might influence plant nutrient allocation strategies. Decreasing temperature shortens the growing season length and leaf lifespan for deciduous broad-leaved plants^34^, hence more nutrients are allocated to leaves to maximize leaf functions for exploiting light resources during the shorter growing season.

In addition, although we did not detail the allocation of C concentrations, as showed in Fig. S3, the scaling exponents of twig stem C to leaf C concentration increased with MAT, suggesting that, as plant C concentration increased, plants at high temperature tended to a larger increase in twig stem C concentration. This further supports that a higher phloem loading and carbonhydrate transport in twig stems occurred for plants at high temperature, resulting in a higher increase in twig stem nutrient concentration.

Precipitation and soil nutrients might be potential drivers for these biogeographical patterns of nutrient allocation, considering their important roles in plant growth and biogeochemical cycling^5^. However, our study found that there were no significant relationships between scaling exponents and AP, soil TN and soil TP (Fig. 3). Eastern China exhibits a steeper thermal gradient than moisture gradient^13^. Given the high soil heterogeneity, soil TN and soil TP might not directly reflect the real nutrient availability for plants. In addition, these environmental factors vary collaterally with each other (Table S4), and their independent roles might be difficult to detect. Thus, future studies are needed to reveal their single effects on plant nutrient allocation strategies. Additionally, there was little impact of altitude on these patterns of nutrient allocation strategies because all scaling exponents showed no significant relationships with altitude (Fig. S4). Changes in the environmental conditions along the altitudinal gradient relative to latitudinal gradient were much smaller (Table S1), and thus the influence of altitude should be covered by that of latitude.

Plant N:P ratio is widely used as an indicator of N and P limitation in terrestrial ecosystem^38^. Schreeg *et al.* (2014)^6^ found that the scaling exponent of stem N:P ratio to leaf N:P ratio for tropical tree seedlings was >1, suggesting that stem N:P ratio might be a better indicator of soil nutrient availability. However, whether this could be applied to other species is little known. In this study, we found that *α*_N:P_ varied among functional groups and along the latitudinal gradient. Woody plants at tropical forests show a higher variability of twig stem N:P ratios with *α*_N:P_ > 1, whereas a more constrained twig stem N:P ratios with *α*_N:P_ < 1 occurred for plants at boreal forests (Fig. 2f). Thus, whether twig N:P or leaf N:P is more sensitive to soil nutrient availability or other factors might depend on functional groups and sites.

Our findings suggest that functional groups and environmental factors jointly influence allocation of nutrients among organs, and result in a gradual shift in nutrient allocation strategy from low to high latitudes across a large geographic scale in eastern China. Variations in the nutrient allocation across functional groups and environmental factors can be explained by the ‘optimal partitioning theory’ or ‘functional equilibrium concept’^22^,^39^, which means that plants would allocate their nutrients in an optimal way to obtain the ‘functional equilibrium’ for fitness. This large-scale pattern is more dependent upon temperature than other environmental factors (i.e., AP, soil TN and soil TP) according to our study, indicating that allocation strategy may also follow the “temperature-plant physiological hypothesis”^27^. Our results reveal that the scaling relationship is more “variable” than “invariant”, subject to the changes in plant nutrient allocation strategy with ambient environment. In general, by exploring the stoichiometric scaling of nutrients between leaves and twigs, we provide a new dimension to understand how plants regulate nutrient allocation strategies to adapt to ambient environment, and thus would broaden our knowledge about the interactions between plants and their environments from a nutrient allocation perspective.

## Materials and Methods

### Study sites

This study was conducted at 12 forest sites across eastern China with latitude from 18.7 °N to 50.9 °N (Fig. S1). Eastern China spans a large range of climates from cold and dry in the north to warm and moist in the south^13^, and has diverse vegetation types from boreal coniferous forest to tropical rainforest. Climate data, including mean annual temperature (MAT) and annual precipitation (AP), were obtained from local reports (Table S1). In the study area, MAT and AP ranges from −5.7 °C to 25.3 °C and from 423 mm to 2031 mm respectively, and soil types shift from nutrient-rich brown soils to nutrient-poor tropical red soils. We classified these 12 sites into three biomes: boreal, temperate and tropical forests^40^. Here, tropical forests included forests situated at 18.7 °N and 23.2 °N, temperate forests included those situated between 29.8 °N and 42.4 °N, boreal forests included those situated between 45.3 °N and 50.9 °N.

### Sampling and Measurement

We collected twig stem and leaf samples at 12 forest sites across eastern China, according to a previous protocol^41^. During the growing season (July–August), we chose dominant or common species at each site, and then collected the fully expanded sun leaves from four or five individuals of each species. Accordingly, terminal 10–20 cm of twig stems (i.e., top twig stems) that supported the sun leaves were sampled. In total, we sampled 335 woody species in 198 genera and 73 families. Samples were dried at 60 °C to constant weight and then powdered using a ball mill (NM200, Retsch, Haan, Germany) before measuring N and P concentrations. Twig stem and leaf N concentrations were determined by the Dumas combustion method using an elemental analyser (2400 II CHS/O, Perkin-Elmer, USA). Twig and leaf P concentrations were determined using the molybdate/ascorbic acid method after H_2_SO_4_-HClO_4_ digestion^42^. For each site, we also collected three soil samples in three plots where these plants were sampled. A horizon samples of soils were randomly sampled and then thoroughly pooled for each plot to represent one soil sample. Soil samples were air-dried, sieved through a 2-mm mesh, handpicked to remove plant detritus, and ground to pass through a 100-mesh sieve. We measured soil total N (TN) and total P (TP) concentrations using the same method for plant samples. Average values of three soil samples at the same site were calculated to represent the soil TN and TP concentration of each site.

### Statistical analysis

Stoichiometric scaling relationships of twig stem N (or P, or N:P ratio) and leaf N (or P, or N:P ratio) were analysed using all original data of N and P concentrations and N:P mass ratios from individual plants (see Appendix S1 for details). Data were log_10_-transformed before analysis. RMA regression was used to determine the scaling function^43^, which was expressed by log_10_ Y = α*(log_10_ X) + *β*, where X and Y represent leaf N (or P, or N:P ratio) and twig stem N (or P, or N:P ratio), and *α* and *β* are the slope (i.e., scaling exponent) and intercept of regression line respectively. We performed these analyses in three ways. First, we divided all original data into respective functional groups (conifer/deciduous broad-leaved/evergreen broad-leaved; legume/non-legume) and three biomes (tropical/temperate/boreal forests), and then compared their scaling exponents. Second, we performed scaling analyses with original data for each site, and then related scaling exponents to the latitude and environmental factors (MAT, AP, soil TN, soil TP) of each site using linear regressions. Third, we compared scaling exponents of evergreen broad-leaved plants from tropical and temperate forests, and scaling exponents of deciduous broad-leaved plants from temperate and boreal forest. A likelihood ratio test was used to indicate the heterogeneity of RMA regression exponents among groups^43^. In addition, linear regressions were used to explore the relationships between log_10_-transformed ratios of leaf N (or P) : twig stem N (or P) and latitude and environmental factors. All statistical analyses were performed using R 2.15.2^44^.

## Additional Information

**How to cite this article**: Yan, Z. *et al.* Nutrient allocation strategies of woody plants: an approach from the scaling of nitrogen and phosphorus between twig stems and leaves. *Sci. Rep.* **6**, 20099; doi: 10.1038/srep20099 (2016).

## Supplementary Material

Supplementary Information

Supplementary Dataset 1

## Figures and Tables

**Table 1:** Reduced major axis (RMA) regression results between twig stem N (or P, or N:P ratio) and leaf N (or P, or N:P ratio) (e.g. log_10_ twig stem N = α*(log_10_ leaf N) + *β*) for all raw data pooled.

	***n***	***α***_**RMA**_ **(95% CI)**	***β***_**RMA**_ **(95% CI)**	***r***^**2**^	***p***
*N*					
All	1513	0.98 (0.94; 1.02)	−0.35 (−0.41; −0.30)	0.28	<0.001
Functional group					
Conifer	84	0.95 a (0.77; 1.16)	−0.24 (−0.46; −0.02)	0.16	<0.001
Deciduous	993	1.19 a (1.13; 1.26)	−0.70 (−0.78; −0.61)	0.32	<0.001
Evergreen	436	1.20 a (1.12; 1.30)	−0.53 (−0.64; −0.42)	0.36	<0.001
Legume	83	1.44 a (1.21; 1.72)	−1.08 (−1.48; −0.69)	0.34	<0.001
Non-legume	1494	0.99 b (0.94; 1.03)	−0.36 (−0.42; −0.30)	0.23	<0.001
*P*					
All	1498	0.89 (0.86; 0.93)	−0.13 (−0.14; −0.12)	0.39	<0.001
Functional group					
Conifer	82	0.70 c (0.59; 0.84)	−0.09 (−0.13; −0.06)	0.37	<0.001
Deciduous	978	0.96 b (0.91; 1.01)	−0.20 (−0.22; −0.18)	0.36	<0.001
Evergreen	438	1.26 a (1.17; 1.35)	−0.00 (−0.02; 0.02)	0.41	<0.001
Legume	83	1.86 a (1.50; 2.29)	−0.33 (−0.41; −0.25)	0.08	0.012
Non-legume	1479	0.88 b (0.85; 0.92)	−0.13 (−0.14; −0.12)	0.40	<0.001
*N:P mass ratio*					
All	1481	1.04 (1.00; 1.08)	−0.28 (−0.33; −0.23)	0.39	<0.001
Functional group					
Conifer	82	0.62 c (0.52; 0.74)	0.23 (0.10; 0.35)	0.35	<0.001
Deciduous	966	0.89 b (0.84; 0.93)	−0.09 (−0.14; −0.04)	0.35	<0.001
Evergreen	433	1.53 a (1.42; 1.64)	−0.95 (−1.09; −0.80)	0.42	<0.001
Legume	83	2.07 a (1.71; 2.51)	−1.68 (−2.23; −1.13)	0.23	<0.001
Non-legume	1462	1.03 b (0.99; 1.07)	−0.27 (−0.32; −0.22)	0.36	<0.001

Different letters indicate significant difference (*p* < 0.05) based on a likelihood ratio test.

## References

[b1] Bazzaz F. A. & Grace J. Plant resource allocation [143–158]. (Academic press, San Diego, 1997).

[b2] Kerkhoff A. J., Fagan W. F., Elser J. J. & Enquist B. J. Phylogenetic and growth form variation in the scaling of nitrogen and phosphorus in the seed plants. Am. Nat. 168, E103–E122 (2006). 17004214 10.1086/507879

[b3] Lambers H., Chapin F. S. & Pons T. L. Plant physiological ecology [255–352]. (Springer-Verlag, New York, 2008).

[b4] Sardans J. & Peñuelas J. Tree growth changes with climate and forest type are associated with relative allocation of nutrients, especially phosphorus, to leaves and wood. Glob. Ecol. and Biogeogr. 22, 494–507 (2013).

[b5] Aerts R. & Chapin F. S. The mineral nutrition of wild plants revisited: a re-evaluation of processes and patterns. Adv. Ecol. Res. 30, 1–67 (2000).

[b6] Schreeg L. A., Santiago L. S., Wright S. J. & Turner B. L. Stem, root, and older leaf N:P ratios are more responsive indicators of soil nutrient availability than new foliage. Ecology 95, 2062–2068 (2014). 25230458 10.1890/13-1671.1

[b7] Yang X. *et al.* Scaling of nitrogen and phosphorus across plant organs in shrubland biomes across Northern China. Sci. Rep. 4 (2014). 10.1038/srep05448 PMC4071319 24965183

[b8] Wright I. J., Reich P. B. & Westoby M. Least-cost input mixtures of water and nitrogen for photosynthesis. Am. Nat. 161, 98–111 (2003). 12650465 10.1086/344920

[b9] Palmroth S. *et al.* On the complementary relationship between marginal nitrogen and water-use efficiencies among Pinus taeda leaves grown under ambient and CO_2_-enriched environments. Ann. Bot. 111, 467–477 (2013). 23299995 10.1093/aob/mcs268 PMC3579436

[b10] Zhu J. L., Shi Y., Fang L. Q., Liu X. E. & Ji C. J. Patterns and determinants of wood physical and mechanical properties across major tree species in China. Science China Life Sciences, 1–11 (2015). 10.1007/s11427-015-4847-y 25921943

[b11] Niklas K. J., Owens T., Reich P. B. & Cobb E. D. Nitrogen/phosphorus leaf stoichiometry and the scaling of plant growth. Ecol. Lett. 8, 636–642 (2005).

[b12] Reich P. B. *et al.* Evidence of a general 2/3-power law of scaling leaf nitrogen to phosphorus among major plant groups and biomes. Proc. R. Soc. B Biol. Sci. 277, 877–883 (2010). 10.1098/rspb.2009.1818 PMC2842731 19906667

[b13] Han W. X., Fang J. Y., Reich P. B., Ian Woodward F. & Wang Z. H. Biogeography and variability of eleven mineral elements in plant leaves across gradients of climate, soil and plant functional type in China. Ecol. Lett. 14, 788–796 (2011). 21692962 10.1111/j.1461-0248.2011.01641.x

[b14] Wright I. J. *et al.* The worldwide leaf economics spectrum. Nature 428, 821–827 (2004). 15103368 10.1038/nature02403

[b15] Reich P. B. *et al.* Scaling of respiration to nitrogen in leaves, stems and roots of higher land plants. Ecol. Lett. 11, 793–801 (2008). 18445031 10.1111/j.1461-0248.2008.01185.x

[b16] Sterner R. W. & Elser J. J. Ecological stoichiometry: the biology of elements from molecules to the biosphere [138–142]. (Princeton University Press, Princeton, 2002).

[b17] Enquist B. J. & Niklas K. J. Global allocation rules for patterns of biomass partitioning in seed plants. Science 295, 1517–1520 (2002). 11859193 10.1126/science.1066360

[b18] Westoby M. & Wright I. J. The leaf size–twig size spectrum and its relationship to other important spectra of variation among species. Oecologia 135, 621–628 (2003). 16228258 10.1007/s00442-003-1231-6

[b19] Sun S. C., Jin D. M. & Shi P. L. The leaf size–twig size spectrum of temperate woody species along an altitudinal gradient: an invariant allometric scaling relationship. Ann. Bot. 97, 97–107 (2006). 16254019 10.1093/aob/mcj004 PMC2803375

[b20] Niklas K. J. *et al.* “Diminishing returns” in the scaling of functional leaf traits across and within species groups. Proc. Natl Acad. Sci. USA 104, 8891–8896 (2007). 17502616 10.1073/pnas.0701135104 PMC1885598

[b21] Pallardy S. G. Physiology of woody plants [19–27]. (Academic Press, London, 2010).

[b22] Poorter H. *et al.* Biomass allocation to leaves, stems and roots: meta-analyses of interspecific variation and environmental control. New Phytol. 193, 30–50 (2012). 22085245 10.1111/j.1469-8137.2011.03952.x

[b23] Yao F. Y. *et al.* Biogeographic patterns of structural traits and C: N: P stoichiometry of tree twigs in China’s forests. PLoS ONE 10, e0116391 (2015). 25664764 10.1371/journal.pone.0116391 PMC4321987

[b24] Marschner H. & Marschner P. Marschner’s mineral nutrition of higher plants [135–158]. (Academic press, London, 2012).

[b25] Marschnert H., Kirkby E. A. & Engels C. Importance of cycling and recycling of mineral nutrients within plants for growth and development. Bot. Acta. 110, 265–273 (1997).

[b26] Reich P. B., Walters M. B. & Ellsworth D. S. From tropics to tundra: Global convergence in plant functioning. Proc. Natl Acad. Sci. USA 94, 13730–13734 (1997). 9391094 10.1073/pnas.94.25.13730 PMC28374

[b27] Reich P. B. & Oleksyn J. Global patterns of plant leaf N and P in relation to temperature and latitude. Proc. Natl Acad. Sci. USA 101, 11001–11006 (2004). 15213326 10.1073/pnas.0403588101 PMC503733

[b28] Han W. X., Fang J. Y., Guo D. L. & Zhang Y. Leaf nitrogen and phosphorus stoichiometry across 753 terrestrial plant species in China. New Phytol. 168, 377–385 (2005). 16219077 10.1111/j.1469-8137.2005.01530.x

[b29] Kattge J. *et al.* TRY-a global database of plant traits. Glob. Change Biol. 17, 2905–2935 (2011).

[b30] Chen Y. H., Han W. X., Tang L. Y., Tang Z. Y. & Fang J. Y. Leaf nitrogen and phosphorus concentrations of woody plants differ in responses to climate, soil and plant growth form. Ecography 36, 178–184 (2013).

[b31] Givnish T. J. Adaptive significance of evergreen vs. deciduous leaves: solving the triple paradox. Silva Fenn. 36, 703–743 (2002).

[b32] Takashima T., Hikosaka K. & Hirose T. Photosynthesis or persistence: nitrogen allocation in leaves of evergreen and deciduous Quercus species. Plant Cell Environ. 27, 1047–1054 (2004).

[b33] Wright I. J. *et al.* Modulation of leaf economic traits and trait relationships by climate. Glob. Ecol. Biogeogr. 14, 411–421 (2005).

[b34] van Ommen Kloeke A. E. E., Douma J. C., Ordoñez J. C., Reich P. B. & Van Bodegom P. M. Global quantification of contrasting leaf life span strategies for deciduous and evergreen species in response to environmental conditions. Glob. Ecol. Biogeogr. 21, 224–235 (2012).

[b35] Vitousek P. M. & Field C. B. Ecosystem constraints to symbiotic nitrogen fixers: a simple model and its implications. Biogeochemistry 46, 179–202 (1999).

[b36] Kerkhoff A. J., Enquist B. J., Elser J. J. & Fagan W. F. Plant allometry, stoichiometry and the temperature‐dependence of primary productivity. Glob. Ecol. Biogeogr. 14, 585–598 (2005).

[b37] Reich P. B. *et al.* Temperature drives global patterns in forest biomass distribution in leaves, stems, and roots. Proc. Natl Acad. Sci. USA 111, 13721–13726 (2014). 25225412 10.1073/pnas.1216053111 PMC4183289

[b38] Güsewell S. N.: P ratios in terrestrial plants: variation and functional significance. New Phytol. 164, 243–266 (2004). 10.1111/j.1469-8137.2004.01192.x 33873556

[b39] Gedroc J. J., McConnaughay K. D. M. & Coleman J. S. Plasticity in root/shoot partitioning: optimal, ontogenetic, or both? Funct. Ecol. 10, 44–50 (1996).

[b40] Yuan Z. Y., Chen H. Y. H. & Reich P. B. Global-scale latitudinal patterns of plant fine-root nitrogen and phosphorus. Nature commun. 2, doi: 10.1038/ncomms1346 (2011). 21673665

[b41] Cornelissen J. H. C. *et al.* A handbook of protocols for standardised and easy measurement of plant functional traits worldwide. Aust. J. Bot. 51, 335–380 (2003).

[b42] John M. K. Colorimetric determination of phosphorus in soil and plant materials with ascorbic acid. Soil Sci. 109, 214–220 (1970).

[b43] Warton D. I., Wright I. J., Falster D. S. & Westoby M. Bivariate line‐fitting methods for allometry. Biol. Rev. Camb. Philos. Soc. 81, 259–291 (2006). 16573844 10.1017/S1464793106007007

[b44] R Development Core Team. R: A Language and Environment for Statistical Computing. (R Foundation for Statistical Computing, Vienna, 2012).

