# Supplementary material for: Nutrient allocation strategies of woody plants: an approach from the scaling of nitrogen and phosphorus between twig stems and leaves
Source: Sci Rep. 2016 Feb 5;6:20099. doi: 10.1038/srep20099 (PMC4742826; doi:10.1038/srep20099)
Supplement: Supplementary Information [file srep20099-s1.doc]

**Supplementary Information**

**Nutrient allocation strategies of woody plants: an approach from the scaling of nitrogen and phosphorus between twig stems and leaves**

Zhengbing Yan^1#^, Peng Li^1#^, Yahan Chen^2^, Wenxuan Han^3^, and Jingyun Fang^1*^

^1^ Department of Ecology, College of Urban and Environmental Sciences, and Key Laboratory for Earth Surface Processes of the Ministry of Education, Peking University, Beijing, China 100871

^2^ Institute of Botany, Chinese Academy of Sciences, Beijing, China 100093

3 College of Resources and Environmental Sciences, Beijing Key Laboratory of Biodiversity and Organic Farming, China Agricultural University, Beijing, China 100193

^*^To whom correspondence should be addressed; E-mail: [jyfang@urban.pku.edu.cn](mailto:jyfang@urban.pku.edu.cn)

**Supplementary Information**

**Supplementary Figures S1-S4**

**Supplementary Tables S1**–**S4**

**Supplementary Figures**


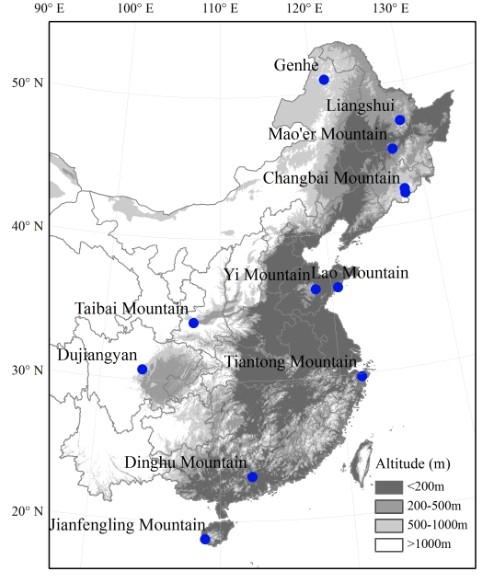
**Figure S1** Distribution of sampling sites from 12 forests across eastern China. This map was created using ArcGIS 10.3 (http://www.esri.com/).


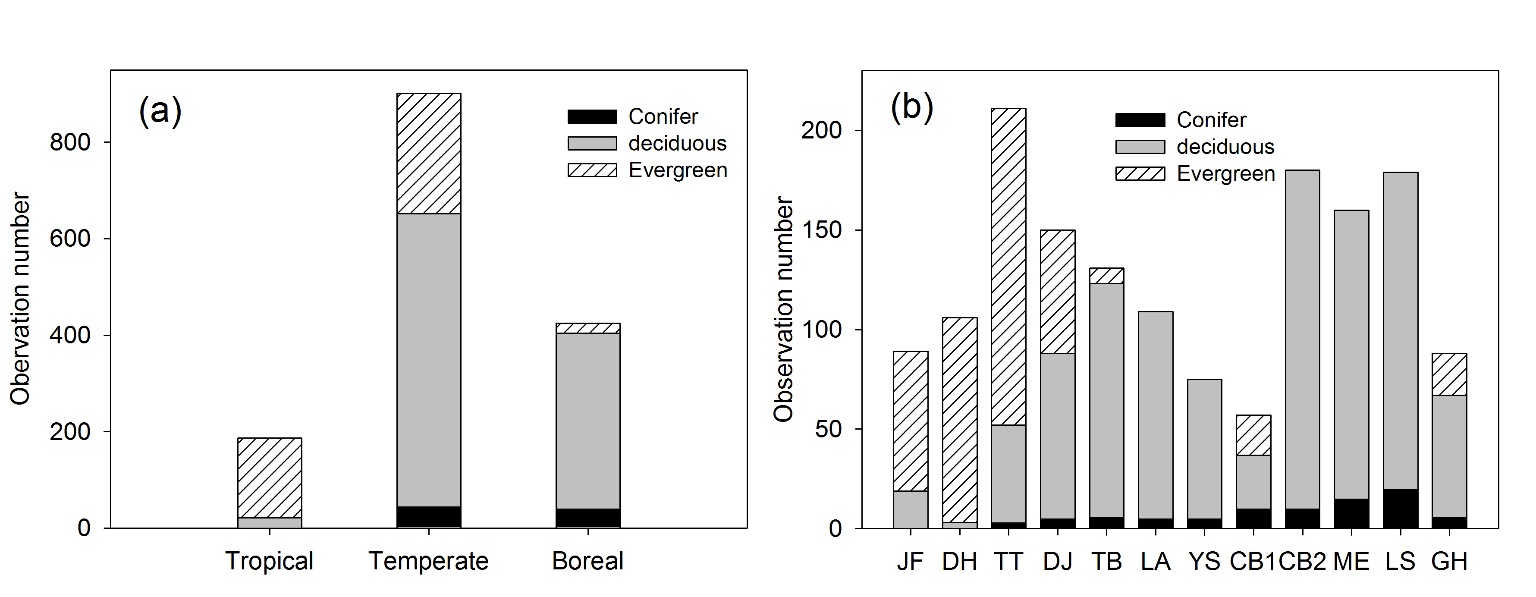
**Figure S2** Species composition at different biomes (tropical, temperate and boreal forests), and at different sites along the increasing latitude.


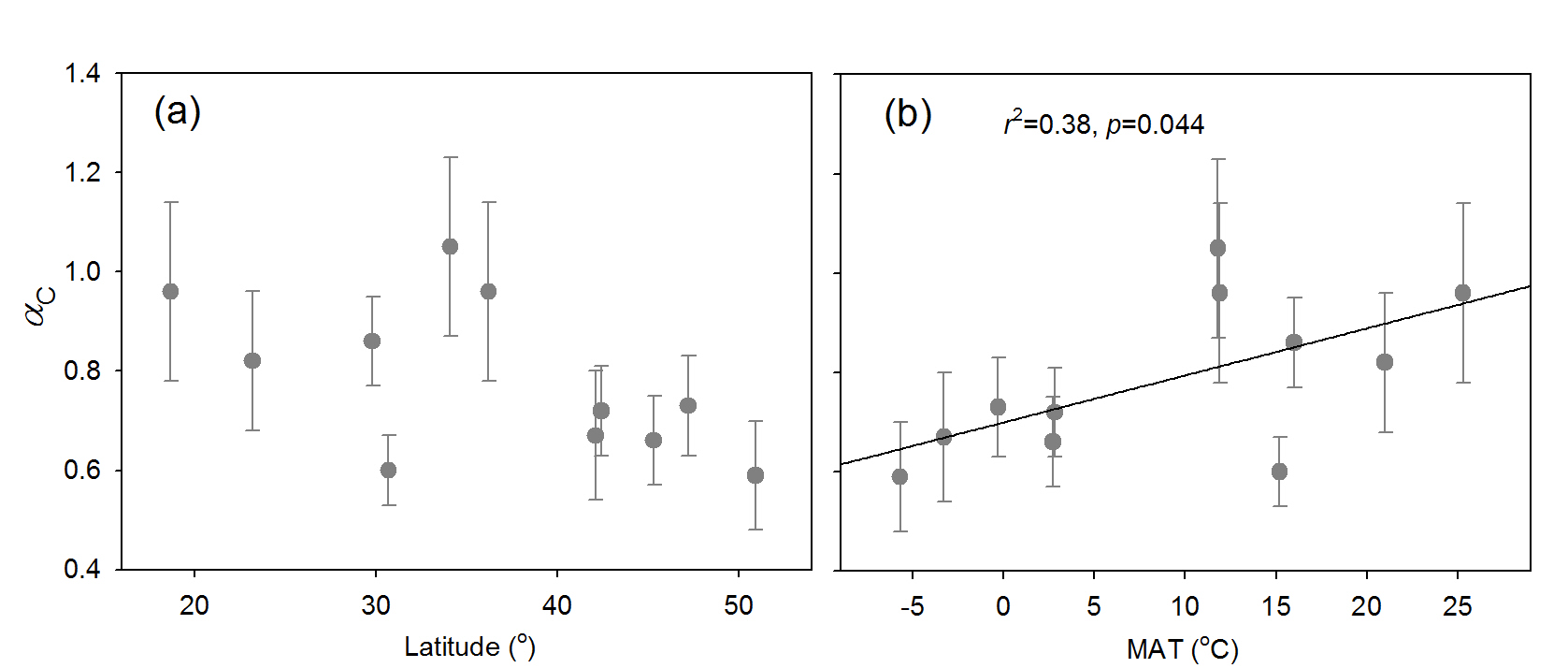
**Figure S3** Relationships between scaling exponent (*α*_C_) and latitude, and MAT. *α*_C_ were determined from the reduce major axis (RMA) regression results between twig stem C and leaf C concentration (i.e. log_10_ twig stem C=*α*_C_***(log_10_ leaf C)+*β*) for all raw data pooled. Significant (*p*<0.05) regression line is fit to the exponents.


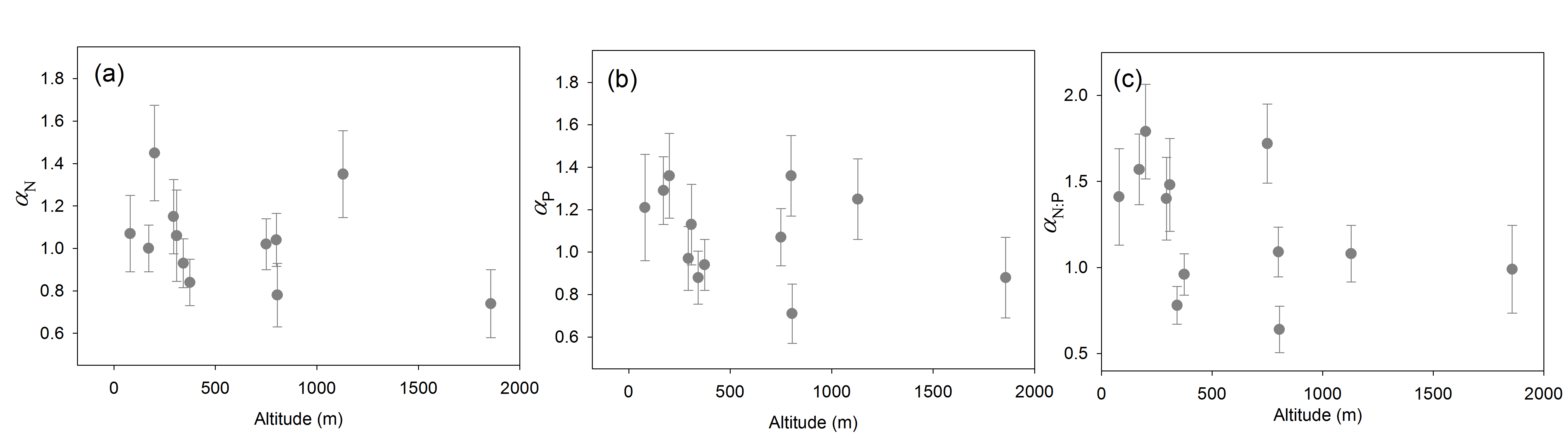
**Figure S4** Relationship between scaling exponents (*α*_N_, *α*_P_ and *α*_N:P_) and altitude. Points and error bars show the exponent and 95% confidence interval (CI). The linear regression equations are not significant with *p*>0.05, indicating the small influence of altitude on these scaling exponents.

**Supplementary Tables**

**Table S1**. Site information. MAT: mean annual temperature, and AP: annual precipitation.

| Site | Latitude  (°N) | Longitude  (°E) | Altitude  (m) | Vegetation type | Biomes | MAT  (°C) | AP  (mm) | Soil TN  (mg g^-1^) | Soil TP  (mg g^-1^) | No. of  species |
| --- | --- | --- | --- | --- | --- | --- | --- | --- | --- | --- |
| Jianfengling (JF) | 18.7 | 108.8 | 80 | Tropical seasonal semi-deciduous forest | Tropical | 25.3 | 2031 | 2.28 | 0.44 | 42 |
| Dinghu Mountain (DH) | 23.2 | 112.5 | 200 | Monsoon evergreen broad-leaved forest | Tropical | 21.0 | 1996 | 1.47 | 0.38 | 40 |
| Tiantong Mountain (TT) | 29.8 | 121.8 | 171 | Subtropical evergreen broad-leaved forest | Temperate | 16.0 | 1551 | 3.10 | 0.41 | 53 |
| Dujiangyan (DJ) | 30.7 | 103.5 | 750 | Subtropical evergreen broad-leaved forest | Temperate | 15.2 | 1244 | 4.00 | 0.46 | 74 |
| Taibai Mountain (TB) | 34.1 | 107.7 | 1129 | Warm temperate deciduous broad-leaved forest | Temperate | 11.8 | 734 | 2.93 | 0.83 | 49 |
| Lao Mountain (LA) | 36.2 | 120.6 | 294 | Warm temperate deciduous broad-leaved forest | Temperate | 11.9 | 743 | 2.25 | 0.50 | 27 |
| Yi Mountain (YS) | 36.2 | 118.6 | 309 | Warm temperate deciduous broad-leaved forest | Temperate | 10.8 | 846 | 4.35 | 0.78 | 22 |
| Changbai Mountain 1 (CB1) | 42.1 | 128.1 | 1857 | Subalpine Betula ermanii forest | Temperate | -3.3 | 1038 | 3.00 | 1.10 | 12 |
| ChangbaiMountain 2 (CB2) | 42.4 | 128.1 | 800 | Broad-leaved Korean pine forest | Temperate | 2.8 | 713 | 6.90 | 1.29 | 40 |
| Mao’er Mountain (ME) | 45.3 | 127.6 | 342 | Secondary deciduous broad-leaved forest | Boreal | 2.7 | 780 | 6.28 | 1.09 | 37 |
| Liangshui (LS) | 47.2 | 128.9 | 374 | Broad-leaved Korean pine forest | Boreal | -0.3 | 680 | 6.85 | 1.12 | 39 |
| Genhe (GH) | 50.9 | 121.5 | 805 | Temperate coniferous forest | Boreal | -5.7 | 427 | 3.28 | 1.14 | 19 |

**Table S2** Summary of reduced major axis (RMA) regression results between twig stem N (or P, or N:P ratio) and leaf N (or P, or N:P ratio) (e.g. log_10_ twig stem N=*α**(log_10_ leaf N)+*β* ) for woody plants grouped by three biomes (tropical, temperate or boreal forests). Different letters indicate significant difference (*p*<0.05) based on a likelihood ratio test.

|  | *n* | *α*_RMA_ (95% CI) | *β*_RMA_ (95% CI) | *r^2^* | *p* |
| --- | --- | --- | --- | --- | --- |
| *N* |  |  |  |  |  |
| Tropical | 187 | 1.30 a (1.16; 1.45) | -0.64 (-0.83; -0.46) | 0.42 | <0.001 |
| Temperate | 901 | 0.97 b (0.92; 1.02) | -0.35 (-0.42; -0.27) | 0.27 | <0.001 |
| Boreal | 425 | 0.89 b (0.82; 0.96) | -0.28 (-0.35; -0.16) | 0.32 | <0.001 |
| *P* |  |  |  |  |  |
| Tropical | 190 | 1.58 a (1.42; 1.76) | 0.01 (-0.03; 0.05) | 0.44 | <0.001 |
| Temperate | 896 | 0.97 b (0.93; 1.02) | -0.12 (-0.13; -0.09) | 0.44 | <0.001 |
| Boreal | 412 | 0.80 c (0.73; 0.87) | -0.19 (-0.23; -0.17) | 0.18 | <0.001 |
| *N:P ratio* |  |  |  |  |  |
| Tropical | 184 | 1.84 a (1.67; 2.02) | -1.33 (-1.56; -1.10) | 0.56 | <0.001 |
| Temperate | 885 | 1.19 b (1.13; 1.26) | -0.51 (-0.59; -0.44) | 0.41 | <0.001 |
| Boreal | 412 | 0.74 c (0.68; 0.80) | 0.14 (0.07; 0.20) | 0.24 | <0.001 |

| Sites | Latitude(^o^) | *n* | *α*_RMA_ (95% CI) | *β*_RMA_ (95% CI) | *r*^2^ | *p* |
| --- | --- | --- | --- | --- | --- | --- |
| N | | | | | | |
| JF | 18.7 | 89 | 1.07 (0.90; 1.26) | -0.37 (-0.59; -0.15) | 0.39 | <0.001 |
| DH | 23.2 | 98 | 1.45 (1.24; 1.69) | -0.84 (-1.13; -0.55) | 0.42 | <0.001 |
| TT | 29.8 | 211 | 1.00 (0.90; 1.12) | -0.29 (-0.43; -0.16) | 0.32 | <0.001 |
| DJ | 30.7 | 148 | 1.02 (0.91; 1.15) | -0.37 (-0.54; -0.20) | 0.45 | <0.001 |
| TB | 34.1 | 128 | 1.35 (1.16; 1.57) | -0.95 (-1.23; -0.66) | 0.25 | <0.001 |
| LA | 36.2 | 104 | 1.15 (0.99; 1.34) | -0.69 (-0.94; -0.45) | 0.39 | <0.001 |
| YS | 36.2 | 75 | 1.06 (0.86; 1.29) | -0.54 (-0.84; -0.24) | 0.23 | <0.001 |
| CB1 | 42.1 | 57 | 0.74 (0.60; 0.92) | -0.03 (-0.25; 0.18) | 0.36 | <0.001 |
| CB2 | 42.4 | 178 | 1.04 (0.92; 1.17) | -0.46 (-0.63; -0.3) | 0.38 | <0.001 |
| ME | 45.3 | 160 | 0.93 (0.82; 1.05) | -0.32 (-0.48; -0.15) | 0.37 | <0.001 |
| LS | 47.2 | 177 | 0.84 (0.74; 0.96) | -0.18 (-0.33; -0.02) | 0.24 | <0.001 |
| GH | 50.9 | 88 | 0.78 (0.64; 0.94) | -0.11 (-0.31; -0.09) | 0.18 | <0.001 |
| P | | | | | | |
| JF | 18.7 | 86 | 1.21 (0.99; 1.49) | 0.12 (0.06; 0.18) | 0.08 | 0.010 |
| DH | 23.2 | 104 | 1.36 (1.18; 1.58) | -0.07 (-0.12; -0.03) | 0.44 | <0.001 |
| TT | 29.8 | 209 | 1.29 (1.14; 1.46) | 0.02 (-0.03; 0.06) | 0.16 | <0.001 |
| DJ | 30.7 | 147 | 1.07 (0.95; 1.22) | -0.03 (-0.06; 0.00) | 0.41 | <0.001 |
| TB | 34.1 | 128 | 1.25 (1.07; 1.45) | -0.23 (-0.29; -0.17) | 0.27 | <0.001 |
| LA | 36.2 | 109 | 0.97 (0.83; 1.13) | -0.19 (-0.22; -0.16) | 0.33 | <0.001 |
| YS | 36.2 | 73 | 1.13 (0.96; 1.34) | -0.22 (-0.24; -0.19) | 0.49 | <0.001 |
| CB1 | 42.1 | 56 | 0.88 (0.71; 1.09) | -0.17 (-0.22; -0.12) | 0.39 | <0.001 |
| CB2 | 42.4 | 174 | 1.36 (1.18; 1.56) | -0.25 (-0.30; -0.20) | 0.11 | <0.001 |
| ME | 45.3 | 155 | 0.88 (0.76; 1.01) | -0.20 (-0.25; -0.15) | 0.18 | <0.001 |
| LS | 47.2 | 169 | 0.94 (0.83; 1.07) | -0.22 (-0.27; -0.18) | 0.29 | <0.001 |
| GH | 50.9 | 88 | 0.71 (0.58; 0.86) | -0.26 (-0.34; -0.19) | 0.15 | <0.001 |
| N:P | | | | | | |
| JF | 18.7 | 86 | 1.41 (1.16; 1.72) | -0.89 (-1.20; -0.57) | 0.18 | <0.001 |
| DH | 23.2 | 98 | 1.79 (1.54; 2.09) | -1.23 (-1.62; -0.85) | 0.41 | <0.001 |
| TT | 29.8 | 209 | 1.57 (1.38; 1.79) | -1.06 (-1.35; -0.77) | 0.09 | <0.001 |
| DJ | 30.7 | 146 | 1.72 (1.51; 1.97) | -1.24 (-1.53; -0.94) | 0.35 | <0.001 |
| TB | 34.1 | 125 | 1.08 (0.93; 1.26) | -0.39 (-0.58; -0.19) | 0.24 | <0.001 |
| LA | 36.2 | 104 | 1.40 (1.18; 1.66) | -0.84 (-1.16; -0.52) | 0.25 | <0.001 |
| YS | 36.2 | 73 | 1.48 (1.24; 1.78) | -0.90 (-1.25; -0.54) | 0.42 | <0.001 |
| CB1 | 42.1 | 56 | 0.99 (0.77; 1.28) | -0.19 (-0.49; 0.11) | 0.11 | 0.014 |
| CB2 | 42.4 | 172 | 1.09 (0.95; 1.24) | -0.34 (-0.51; -0.17) | 0.21 | <0.001 |
| ME | 45.3 | 155 | 0.78 (0.68; 0.9) | 0.05 (-0.07; 0.17) | 0.18 | <0.001 |
| LS | 47.2 | 169 | 0.96 (0.85; 1.09) | -0.11 (-0.24; 0.03) | 0.31 | <0.001 |
| GH | 50.9 | 88 | 0.64 (0.52; 0.79) | 0.3 (0.17; 0.41) | 0.05 | 0.046 |

**Table S3** Summary of RMA regression results between twig stem N (or P, or N:P) and leaf N (or P, or N:P) (e.g. log_10_ twig stem N=α*(log_10_ leaf N)+β ) among different sites.

**Table S4** Correlations among latitude and environmental factors. Pearson coefficients and *p* values are shown in the lower left and upper right sections, respectively. MAT, mean annual temperature; AP, annual precipitation; soil TN, soil total nitrogen concentration; soil TP, soil total phosphorus concentration.

|  | Latitude | MAT | AP | Soil TN | Soil TP |
| --- | --- | --- | --- | --- | --- |
| Latitude |  | <0.001 | <0.001 | 0.028 | <0.001 |
| MAT | -0.97 |  | 0.001 | 0.067 | <0.001 |
| AP | -0.90 | 0.82 |  | 0.066 | 0.005 |
| Soil TN | 0.63 | -0.54 | -0.55 |  | 0.009 |
| Soil TP | 0.87 | -0.90 | -0.75 | 0.71 |  |
